# Supplementary material for: Expression of Microtubule-Associated Proteins in Relation to Prognosis and Efficacy of Immunotherapy in Non-Small Cell Lung Cancer
Source: Front Oncol. 2021 Oct 1;11:680402. doi: 10.3389/fonc.2021.680402 (PMC8517487; doi:10.3389/fonc.2021.680402)
Supplement: Supplementary file 1 [file DataSheet_1.zip › immunephenotype/IPS_TCGA-55-6975-11A-01R-1949-07.pdf]

Immunophenoscore: 6

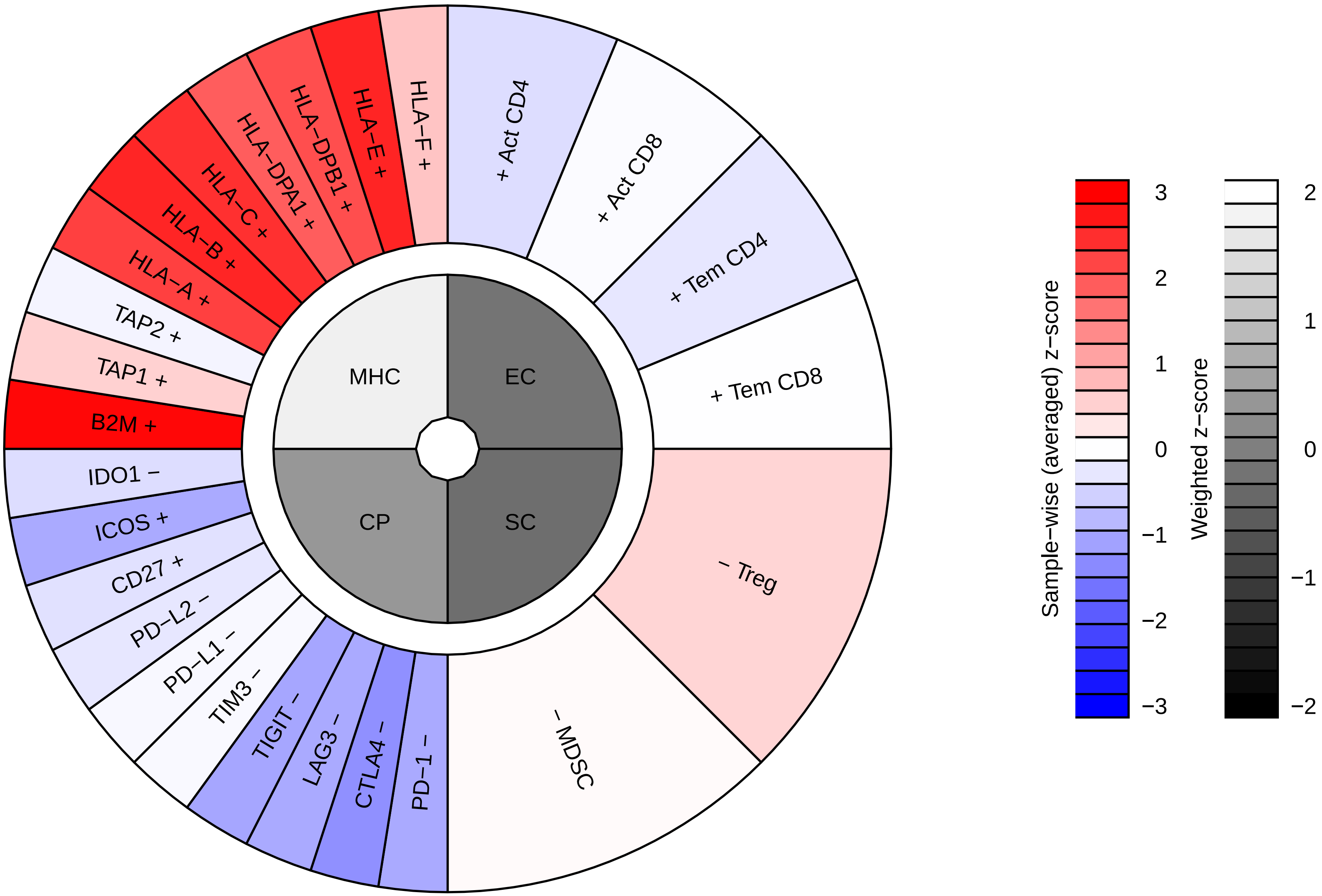

MHC: Antigen Processing

CP: Checkpoints | Immunomodulators

EC: Effector Cells

SC: Suppressor Cells
